# 4D Printing in Regenerative Medicine: Bio-Inspired Applications for Dynamic Tissue Repair

**DOI:** 10.3390/jfb17020072

**Published:** 2026-02-01

**Authors:** Guanyi Liu, Jinan Wu, Yang Yang, Junsi Luo, Xiaoli Xie

**Affiliations:** 1 Xiangya Stomatological Hospital and Xiangya School of Stomatology, Central South University, Changsha 410008, China; 236812032@csu.edu.cn (G.L.); 236812030@csu.edu.cn (J.W.); 236812044@csu.edu.cn (Y.Y.); 2 Hunan 3D Printing Engineering Research Center of Oral Care, Central South University, Changsha 410008, China

**Keywords:** 4D printing, bio-inspired design, regenerative medicine, tissue engineering, stimuli-responsive materials

## Abstract

4D printing, as an advanced evolution of 3D bioprinting, introduces time as an active design dimension, enabling printed constructs to undergo programmed morphological or functional transformations in response to external or endogenous stimuli. By integrating stimuli-responsive smart materials with precise additive manufacturing, 4D printing provides a bio-inspired strategy to overcome the inherent limitations of static scaffolds and to achieve spatiotemporal dynamic matching with the evolving biological microenvironment during tissue regeneration. Over the past decade, significant progress has been made in applying 4D printing to structurally and functionally complex tissues, including bone, muscle, vasculature, nerve repair, wound closure, and other emerging biomedical scenarios. Rather than emphasizing shape change alone, recent advances demonstrate that 4D-printed constructs can emulate key biological processes such as morphogenesis, contraction, directional guidance, electrophysiological signaling, and microenvironment-responsive regulation, thereby enhancing tissue integration and functional recovery. This review systematically summarizes materials, stimulus–response mechanisms, and representative applications of 4D printing from a bio-inspired perspective, while critically discussing current challenges related to material performance, mechanistic understanding, manufacturing precision, and clinical translation. Finally, future perspectives are outlined, highlighting the importance of interdisciplinary integration, intelligent manufacturing, and clinically oriented evaluation frameworks to advance 4D printing toward personalized and precision regenerative medicine.

## 1. Introduction

The rising incidence of age-related degeneration and trauma-associated disorders has substantially increased the clinical demand for repairing injuries to tissues such as bone, muscle, and nerves. Although current mainstream strategies—including autografts/allografts, metal or ceramic implants, tissue-engineered scaffolds, and growth factor- or cell-based therapies—have achieved partial success, several persistent limitations remain. These include restricted donor availability, surgical morbidity and immune rejection, long-term mechanical mismatch, and the difficulty of simultaneously restoring tissue morphology and function. Moreover, therapeutic efficacy is often compromised in complex and dynamically changing physiological microenvironments, where tissue repair follows tightly regulated, stage-dependent biological processes. Collectively, achieving spatiotemporal “dynamic matching” between repair constructs and the evolving regenerative process remains one of the central challenges in regenerative medicine [1,2].

The integration of additive manufacturing with biomedicine has enabled 3D printing to fabricate complex architectures and multimaterial constructs with high geometric precision through extrusion, bioprinting, photopolymerization, and digital light processing (DLP). These advances have significantly improved customization and structural controllability in tissue engineering. However, conventional 3D-printed scaffolds are intrinsically static and therefore poorly suited to accommodate the biochemical, mechanical, and cellular dynamics inherent to tissue regeneration. As a result, their ability to maintain long-term functional compatibility with living tissues is limited, constraining further improvements in biomimetic performance [3,4,5].

In contrast, 4D printing—enabled by smart materials and programmable structural design—represents a transition from static fabrication toward dynamic responsiveness. Rather than focusing solely on material novelty, the significance of 4D printing in regenerative medicine lies in its support of bio-inspired application strategies, in which printed constructs are designed to adapt their morphology, mechanics, or function in response to environmental cues, analogous to adaptive behaviors observed in native tissues. Compared with conventional treatments and static 3D-printed constructs, 4D printing enables enhanced adaptability to physiological changes, sequential or phase-dependent functionality, and improved precision matching during tissue repair.

By integrating materials responsive to stimuli such as temperature, light, magnetic fields, electric fields, humidity, pH, enzymatic activity, or cellular traction, 4D printing enables programmable shape morphing, mechanical tuning, and functional transformation in response to endogenous or exogenous cues. From an application-oriented perspective, these capabilities allow constructs to actively interact with the regenerative microenvironment rather than passively occupy defect sites, thereby supporting dynamic tissue repair processes [6,7,8]. In skeletal tissue regeneration, stimulus-responsive 4D-printed scaffolds can conform to irregular bone or cartilage defects while dynamically adjusting internal architecture during healing, promoting osteogenic and chondrogenic regeneration [9,10]. In vascular repair, hydration-, thermal-, or light-triggered activation enables planar constructs to self-assemble into luminal or branched geometries, facilitating rapid anastomosis and perfusable network formation [11]. In neural regeneration, adaptive conduits can modulate lumen geometry and alignment in response to tissue tension, reducing mechanical mismatch while providing supportive electro- or magneto-responsive microenvironments for axonal growth [12]. Likewise, in muscle and skin repair, 4D-printed scaffolds enable phase-dependent regulation of bioactive factor release and tissue organization, supporting coordinated inflammation control, angiogenesis, and functional tissue remodeling [13].

A further advantage of 4D printing lies in the integration of preoperative digital modeling with intraoperative and post-implantation adaptability. Patient-specific imaging data can be combined with programmable construct behavior to enable secondary morphological or functional adaptation in vivo, achieving high-fidelity matching in geometry, mechanics, and biological performance over time. Consequently, 4D printing has demonstrated superior regenerative potential across multiple tissue types—including bone, cartilage, vascular, neural, muscle, and skin—relative to static 3D-printed approaches [10,14].

Despite rapid progress, most existing reviews emphasize material development or isolated stimulus–response mechanisms, often fragmenting applications by tissue type and lacking a unifying perspective that connects regenerative biology with adaptive repair strategies. To address this gap, the present review summarizes recent advances in 4D printing for representative tissues—including bone, cartilage, vasculature, nerve, muscle, and skin—from the perspective of bio-inspired applications for dynamic tissue repair. While materials and stimulus mechanisms are introduced as enabling tools, emphasis is placed on how 4D printing strategies functionally emulate adaptive behaviors of living tissues to achieve spatiotemporal coordination between constructs and regenerative processes. Common challenges, tissue-specific considerations, and future translational pathways are also discussed to clarify the role of 4D printing in advancing adaptive regenerative therapies.

## 2. 4D Printing Materials and Stimuli-Responsive Mechanisms

The realization of dynamic functionalities in 4D printing is fundamentally governed by the selection of smart materials and their capacity to respond predictably to external stimuli. To date, a wide range of material systems—including shape-memory alloys, ceramics, polymers, hydrogels, and liquid crystalline elastomers—have been integrated into 4D-printed constructs, owing to their inherent ability to undergo stimulus-triggered transformations. Beyond simple shape recovery, these materials can exhibit self-assembly, self-healing, and reversible deformation behaviors, while also enabling biologically relevant shape changes through pre-programmed geometric and structural design. Such characteristics substantially broaden the applicability of 4D printing across tissue-specific regenerative scenarios, where controlled morphological and functional evolution is required [15,16,17]. As the central driving force distinguishing 4D printing from conventional additive manufacturing, dynamic responsiveness shifts the fabrication paradigm from passive, static structures toward active systems capable of functional adaptation over time. With the rapid advancement of intelligent material design and cross-disciplinary integration, the mechanisms governing responses to physical and chemical stimuli—such as temperature, electric fields, light, and related cues—have become increasingly refined and controllable. Collectively, these developments provide the essential technological foundation for constructing complex, spatiotemporally evolving systems that underpin the dynamic capabilities of 4D printing.

### 2.1. Materials for 4D Printing

From a materials perspective, four-dimensional (4D) printing involves the use of printable material systems that can undergo programmable changes in shape, structure, or properties in response to external stimuli. In biomedical applications, such materials are expected to combine processability, controllable responsiveness, mechanical compatibility, and biological functionality. A wide range of material systems has therefore been explored, including polymers, hydrogels, metals, ceramics, and their composites, each contributing distinct physicochemical properties and functional advantages.

#### 2.1.1. Shape Memory Polymers

Shape memory polymers (SMPs) represent one of the most extensively investigated material platforms for 4D printing. Their shape-memory behavior arises from the synergistic interaction between rigid segments, which define the permanent shape, and soft segments, which enable reversible deformation through chain mobility and phase transitions under external stimuli such as temperature, light, electric fields, or chemical environments [18,19,20,21,22,23,24]. Owing to their flexible processability, tunable transition temperatures, lightweight nature, and compatibility with functional fillers, SMPs are particularly well suited for fabricating deployable scaffolds, minimally invasive implants, and programmable drug delivery systems [25,26,27,28,29,30].

#### 2.1.2. Stimuli-Responsive Hydrogels

Stimuli-responsive hydrogels constitute another major class of materials for 4D printing, characterized by three-dimensional hydrophilic polymer networks capable of reversible swelling and shrinkage in response to environmental cues such as temperature, pH, or light [31,32]. Their high water content, excellent biocompatibility, and ability to encapsulate cells or bioactive agents render hydrogels particularly attractive for soft tissue engineering, wound dressings, and smart drug delivery applications [33,34,35]. In biomedical 4D printing, hydrogel-based constructs enable dynamic volumetric and structural adaptation, allowing printed architectures to undergo post-fabrication shape transformation and microenvironment-responsive regulation [36,37,38].

#### 2.1.3. Liquid Crystal Materials

Liquid crystal materials, including liquid crystal polymers (LCPs) and liquid crystal elastomers (LCEs), represent a unique class of smart materials for 4D printing, in which macroscopic deformation is governed by stimulus-induced molecular alignment. These materials integrate anisotropic liquid crystalline mesogens within polymeric or elastomeric networks, enabling large, reversible, and programmable shape changes under thermal, optical, or electrical stimulation. Compared with conventional shape memory materials, liquid crystal systems offer continuous actuation and precise control through molecular orientation programming. Such characteristics make LCPs and LCEs particularly attractive for 4D-printed constructs requiring dynamic motion, including artificial muscles, soft robotics, microscale actuators, and flexible or wearable electronic devices [39,40].

#### 2.1.4. Shape Memory Alloys

Shape memory alloys (SMAs), typified by NiTi-based systems, exhibit reversible martensitic–austenitic phase transformations that enable temperature- or stress-induced shape recovery and superelasticity [41]. Advances in metal additive manufacturing techniques, including laser-based printing strategies, have enabled the fabrication of SMA structures with programmable deformation for applications such as self-expanding stents and adaptive orthopedic implants [42,43,44]. However, high processing temperatures, stiffness mismatch with soft tissues, and limited control over activation conditions remain significant challenges for their broader application in biomedical 4D printing [43,45,46].

#### 2.1.5. Shape Memory Ceramics

Shape memory ceramics (SMCs) rely on reversible crystalline phase transitions to achieve stimulus-induced shape recovery and are typically processed as ceramic-based composites or functional slurries in 4D printing [47,48]. While their exceptional thermal stability and mechanical robustness make them promising for extreme-environment applications, biomedical exploration of SMCs remains at an early stage due to processing complexity, brittleness, and limited biological validation.

#### 2.1.6. Bioactive Inorganic and Natural Materials

Bioactive inorganic materials, particularly hydroxyapatite (HAp) and calcium phosphate–based materials, are widely employed in regenerative medicine owing to their chemical similarity to the mineral phase of native bone and excellent osteoconductivity. Although these materials generally lack intrinsic shape-memory behavior or programmable deformability, their integration into polymeric or hydrogel-based matrices is commonly adopted to enhance the biological and mechanical performance of 4D-printed constructs [49,50]. In regenerative applications, HAp- and phosphate-containing composites are primarily used in bone and dental tissue engineering, where they support cell adhesion, osteogenic differentiation, and interfacial integration while complementing the stimuli-responsive behavior of the polymeric framework [51,52].

Similarly, natural materials, including collagen, gelatin, alginate, chitosan, and hyaluronic acid, are extensively used in regenerative medicine owing to their excellent biocompatibility, biodegradability, and inherent bioactivity. Although most natural polymers lack intrinsic shape-memory behavior, their incorporation into polymeric matrices or crosslinked hydrogel networks enables stimulus-responsive deformation and dynamic shape adaptation [53]. In regenerative applications, these materials are widely employed in cell-laden scaffolds, wound dressings, and soft tissue engineering constructs, where they provide a favorable microenvironment for cell adhesion, proliferation, and tissue regeneration while preserving dynamic structural functionality [54,55].

Together, the representative materials used in biomedical 4D printing, along with their mechanistic properties, key features, and typical applications, are summarized in Table 1.

### 2.2. Stimuli-Responsive Mechanisms in 4D Printing

While Section 2.1 introduces the major material classes used in 4D printing, this section focuses on the stimulus-responsive mechanisms that drive time-dependent shape transformation and functional adaptation. Stimuli responsiveness is the defining feature that distinguishes 4D printing from traditional 3D printing. By endowing materials with the ability to perceive and react to environmental cues, 4D-printed constructs can undergo controllable, time-dependent morphological or functional transformations. Common stimuli include temperature, pH, magnetic fields, light, electric fields, and mechanical stress, each of which modulates molecular conformation, swelling behavior, or phase transitions to drive pre-programmed deformation or functional conversion. Collectively, these multisensory response mechanisms expand the scope of 4D printing in tissue engineering and regenerative medicine, enabling the design of programmable, biomimetic, and adaptively responsive medical materials.

#### 2.2.1. Temperature

Temperature is the most widely utilized stimulus in 4D printing, owing to its direct relevance to physiological environments and the well-established thermal responsiveness of many polymeric systems. Thermo-responsive materials typically undergo controllable shape or volume changes through mechanisms such as phase transitions, crystallization–melting processes, or reversible swelling–shrinkage behavior, enabling predictable actuation under thermal stimuli. For example, poly(ε-caprolactone) (PCL) blended with polyethylene-octene copolymer (POE) exhibits pronounced shape-memory behavior within the range of 55–60 °C, with tunable fixation and recovery ratios as well as stable cyclic performance, making it suitable for thermally activated scaffold designs [56].

Thermo-responsive hydrogels further expand temperature-based actuation strategies by enabling volume-driven deformation under mild thermal variation. Poly(N-isopropylacrylamide) (pNIPAm) hydrogels swell below approximately 32 °C and contract above this threshold, allowing post-implantation self-fitting to complex soft-tissue contours—including cartilage, vasculature, and skin—which improves local microenvironmental compatibility and supports tissue regeneration [57]. Similarly, hydrogels based on poly(acrylic acid) (PAAc) and cetyl acrylate (C16A) exhibit shape-memory and self-healing behavior near physiological temperature, providing a means for dynamically modulating scaffold integrity and tissue healing processes during regeneration [58].

#### 2.2.2. pH

pH-responsive materials are capable of sensing local acid–base fluctuations and enabling on-demand release of therapeutic agents in response to environmental changes. Hydrogels derived from gelatin, poly(2-vinylpyridine) (P2VP), and acrylic acid (AA) can undergo pH-dependent variations in swelling behavior and morphological configuration [59,60,61]. During tissue repair, such responsiveness allows selective delivery of bioactive molecules within pathological microenvironments—such as acidic wounds or inflamed tissues—thereby enhancing cell proliferation, guiding differentiation, modulating inflammatory responses, and ultimately accelerating regenerative processes.

#### 2.2.3. Magnetic Field

Magnetic-responsive materials enable remote, non-contact actuation, offering distinct advantages for implantable systems and targeted therapeutic manipulation. By incorporating magnetic components such as Fe_3_O_4_ nanoparticles, NdFeB particles, or Fe microparticles into polymer matrices, constructs can undergo magnetically driven deformation, movement, or reconfiguration under external magnetic fields [62,63]. For example, PDMS/Fe composite systems exhibit rapid and reversible shape changes upon magnetic stimulation [64], providing dynamically adjustable mechanical support and enabling directional guidance of cell migration, which is beneficial for regenerative applications.

#### 2.2.4. Light

Light-responsive materials provide high spatial and temporal precision, allowing localized activation and fine-tuned modulation of material behavior and the cellular microenvironment. Polymers integrated with photothermal agents such as gold nanoparticles (AuNPs) or photochromic molecules, including azobenzene, can undergo controlled shape recovery or molecular rearrangement upon exposure to specific wavelengths of light [65,66,67]. Owing to the non-contact and programmable nature of optical stimulation, light-based activation enables precise regulation within complex wound sites or engineered tissue constructs, thereby supporting controlled cell organization and functional tissue formation.

#### 2.2.5. Electric Field

Electric field-responsive materials can directly interact with endogenous or externally applied bioelectrical signals, making them particularly relevant for regenerating electrically active tissues such as neural or cardiac systems. Conductive polymers such as polypyrrole (PPy) exhibit volumetric or conformational changes under electrical stimulation, while conductive hydrogels and electroactive composites can transmit electrical cues to guide directional cell growth and promote functional recovery [68]. In addition, Joule heating generated by electrical currents can activate thermo-responsive polymers, triggering rapid and reversible shape transformation. This electrically induced actuation strategy is especially attractive for artificial muscle systems and dynamically tunable scaffolds requiring precise temporal control [69].

#### 2.2.6. Mechanical Stress

Mechanical stress–responsive materials can convert externally applied or physiologically generated mechanical loading into reversible deformation or functional adaptation [41]. For example, NiTi-based SMA structures exhibit reversible deformation and recovery in response to applied stress, allowing self-expanding or load-adaptive behavior that is advantageous for implants operating in dynamic, load-bearing environments [42]. Such mechanical stress responsiveness enables 4D-printed constructs to better accommodate tissue motion and mechanical constraints, thereby improving structural conformity and functional stability during regeneration.

In summary, the diverse stimuli-responsive characteristics of materials employed in 4D printing enable dynamically controllable structural and functional transformations with high biological relevance. By regulating scaffold morphology, conformability, drug release profiles, and cellular behavior through stimuli such as temperature, pH, magnetic fields, light, electrical inputs, and mechanical stress. 4D printing provides a versatile technological framework for modulating regenerative microenvironments, accelerating tissue repair processes, and supporting individualized therapeutic precision.

### 2.3. Printing Techniques in 4D Printing

In biomedical four-dimensional (4D) printing, dynamic functionality is achieved by integrating stimuli-responsive materials with established additive manufacturing techniques. From a technological perspective, current 4D printing approaches are primarily built upon established 3D printing techniques, including extrusion-based methods (e.g., direct ink writing (DIW) and fused deposition modeling (FDM)), inkjet printing, photopolymerization-based techniques (e.g., stereolithography (SLA) and digital light processing (DLP)), and powder-based processes (e.g., selective laser sintering (SLS) and selective laser melting (SLM)). The representative 4D printing techniques and their key characteristics, including processing principles, material compatibility, and typical applications, are summarized in Table 2.

## 3. Bio-Inspired Applications of 4D Printing in Regenerative Medicine and Tissue Engineering

### 3.1. Bone Regeneration: Macro- and Micro-Scale Functional Adaptation

Although the human body possesses regenerative potential across various tissues and organs, the self-repair capacity of the skeletal system remains limited when confronted with large-scale or complex bone defects. Current clinical strategies primarily rely on autologous or allogeneic bone grafting to achieve structural and functional reconstruction through bone substitutes. However, these approaches are constrained by donor site morbidity, limited graft availability, and the risk of immune rejection [79,80]. In addition, bone regeneration outcomes are strongly influenced by multiple interacting factors, including defect geometry, local vascularization, soft tissue condition, infection status, and host systemic factors such as metabolic disease and comorbidities. Together, these variables constitute the major clinical challenges in bone regeneration therapy [81,82].

From a bio-inspired application perspective, 4D-printed scaffolds address these challenges by enabling spatiotemporally programmed adaptation of both macroscopic geometry and microscopic architecture, thereby functionally approximating the dynamic remodeling behavior of native bone tissue [83]. Through controlled shape transformation and microenvironmental regulation, 4D-printed constructs overcome the inherent limitations of static scaffolds and provide a dynamic interface capable of coordinating with the evolving stages of bone healing.

#### 3.1.1. Macroscopic Shape Adaptation for Irregular Bone Defects

**Figure 1:**
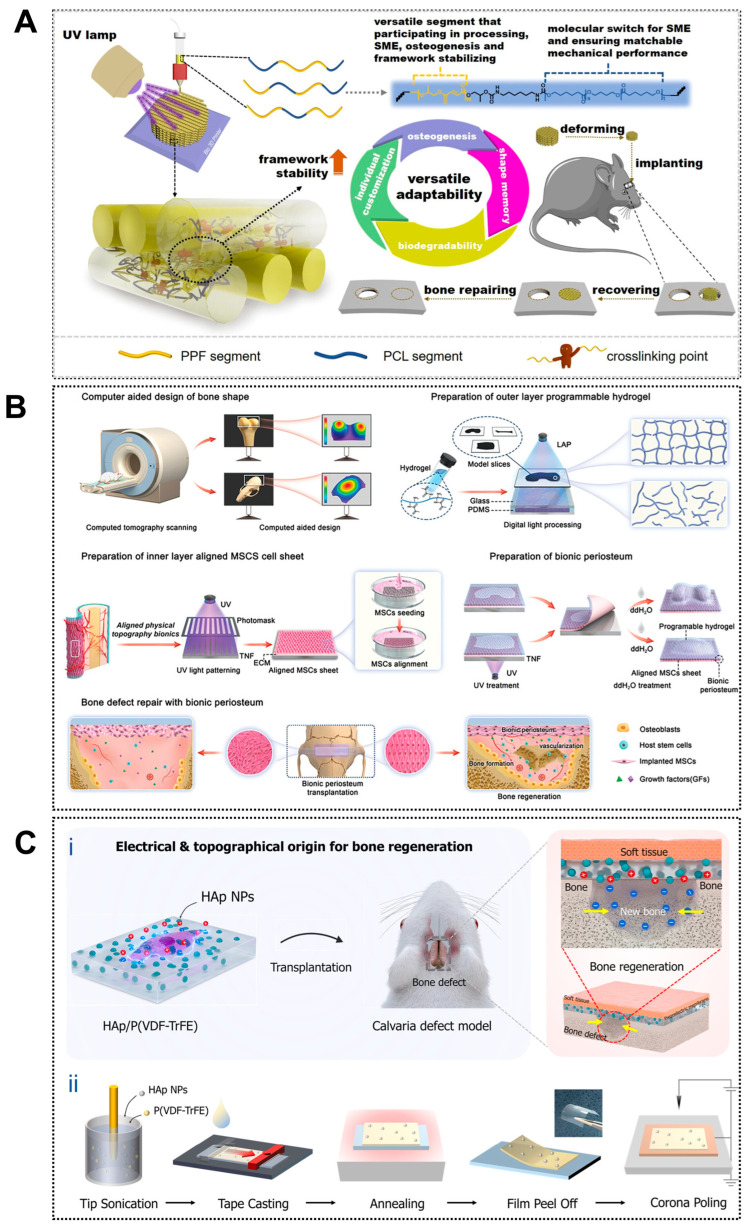
Applications of 4D printing in bone regeneration. (**A**) Design of the biodegradable polyester copolymer and fabrication strategy of the scaffold. Adapted with permission from Ref. [84]. Copyright 2023, American Chemical Society. (**B**) Schematic illustration of fabricating biomimetic periosteum for bone regeneration. Adapted with permission from Ref. [88]. Copyright 2023, Wiley-VCH. (**C**) Design of piezoelectrically and topographically originated biomimetic scaffolds. (**Ci**) Schematic representation of the enhanced bone regeneration mechanism through electrical and topographical cues provided by HAp-incorporated P(VDF-TrFE) scaffolds. (**Cii**) Schematic diagram of the fabrication process. Adapted from [89], under a CC-BY 4.0 licence.

At the macroscopic level, 4D-printed scaffolds enable dynamic and spatiotemporally controlled matching between scaffold configurations and irregular bone defect geometries, offering clear advantages over conventional static 3D-printed constructs. Through programmed actuation, these scaffolds undergo predefined morphological changes in response to physiological or externally applied stimuli. For example, shape-memory polyester scaffolds exhibit body-temperature-activated shape recovery, allowing adaptive fitting within irregular bone defects while maintaining a mechanically stable porous architecture that supports robust new bone formation and mechanical restoration in vivo (Figure 1A) [84]. Similarly, composite scaffolds incorporating photothermal agents such as polydopamine nanoparticles enable temporary compression during implantation, followed by rapid and high-fidelity shape recovery (>99% within 30 s) under near-infrared irradiation, facilitating minimally invasive placement in complex defect sites [85]. Thermally responsive multi-material scaffolds composed of PLA/PCL/β-TCP/Mg further demonstrate volumetric expansion or shape recovery at elevated temperatures for precision defect filling, while simultaneously supporting photothermal tumor ablation and M2-type immune modulation under mild hyperthermia (42 °C), thereby promoting bone regeneration [86]. Beyond polymer-based systems, gradient cross-linked hydrogels have also shown spontaneous, stimulus-induced bending within tunable deformation ranges, translating into enhanced bone formation in vivo [87]. Collectively, these studies demonstrate the ability of 4D-printed scaffolds to achieve multiscale deformation and macroscopic conformity to irregular bone defects, thereby improving in vivo regenerative outcomes.

#### 3.1.2. Microstructural Reconfiguration and Cell–Matrix Interaction

Building upon macroscopic adaptability, 4D printing further enables bio-inspired microstructural reconfiguration that dynamically regulates cell behavior and osteogenic responses. Shape-memory composite scaffolds have been shown to undergo localized changes in pore size, fiber alignment, and surface roughness under stimulation, increasing integrin-binding availability and enhancing nutrient and oxygen diffusion throughout the construct [90]. Patterned printing strategies have enabled the fabrication of anisotropic microarchitectures within shape-memory polymer layers, allowing reversible deformation and dynamic guidance of cell alignment and differentiation (Figure 1B) [88]. These dynamically adjustable microenvironments promote osteogenic differentiation of human mesenchymal stem cells in vitro and accelerate new bone formation in vivo, highlighting the potential of 4D printing for temporally controlled regulation of bone repair processes.

#### 3.1.3. Bio-Inspired Microenvironment for Osteogenesis

The osteogenic performance of 4D-printed scaffolds can be enhanced by recreating a bio-inspired bone microenvironment that improves the biological functionality of conventional 3D-printed constructs and actively drives stem cell osteogenesis. Native bone exhibits a hierarchical architecture with anisotropic mechanical and electromechanical properties that regulate bone remodeling [91]. Inspired by this feature, piezoelectric biomaterials such as barium titanate can generate endogenous electrical signals under mechanical stimulation, thereby promoting osteoblast proliferation and differentiation [92]. Joo et al. further combined hydroxyapatite nanoparticles with the piezoelectric polymer P(VDF-TrFE) to fabricate an electrically active nanostructured scaffold (Figure 1C), which produced stable electrical signals during deformation and effectively simulated the bone electrophysiological microenvironment, enhancing osteogenic activity [89]. These advantages highlight a promising direction for 4D bioprinting through post-printing modulation of piezoelectric effects to improve bone defect repair.

#### 3.1.4. Extension Toward Cartilage and Osteochondral Repair

Beyond bone regeneration, the adaptability and stimulus-responsiveness of 4D-printed constructs also demonstrate strong translational potential in cartilage repair, where minimally invasive deployment and in situ adaptation are increasingly emphasized. Shape-memory polyurethane scaffolds reinforced with nano-hydroxyapatite and designed with “root-like” anchoring architectures inspired by mangrove structures allow compression into minimally invasive forms at room temperature followed by rapid shape recovery at physiological temperature, enabling automatic unfolding after implantation [93]. Injectable PEGDA-based micro-scaffolds fabricated via digital light processing further exhibit spatial folding and autonomous reorganization in response to optical or humidity cues, facilitating in situ reconstruction during arthroscopic procedures while maintaining high cell-loading efficiency and nutrient transport capacity [94]. Magneto-responsive silk-fibroin/gelatin composite constructs additionally enable controlled transitions from planar sheets to three-dimensional curved structures under magnetic fields, allowing precise adaptation to irregular intra-articular lesion contours and promoting chondrocyte adhesion, proliferation, and differentiation [95]. Together, these examples underscore the growing translational readiness of 4D-printed scaffolds for patient-tailored cartilage regeneration.

Overall, these advances position 4D printing as a transformative strategy in bone tissue engineering, enabled by its dual capabilities for spatiotemporal dynamic matching and functional microenvironment reconstruction. Compared with conventional 3D printing, 4D-printed scaffolds not only provide intraoperative shape adaptability but also enable programmable, stimulus-guided structural modulation within physiological environments. The integration of multifunctional features—including piezoelectricity, photothermal responsiveness, and immunomodulatory effects—further allows engineered constructs to more closely approximate native tissue behavior, shifting 4D printing from a purely structural repair approach toward a multifunctional therapeutic platform. Nevertheless, significant challenges remain for clinical translation. Scaffold deformation often depends on external stimuli, raising concerns regarding controllability, uniformity, and in vivo safety, particularly with respect to thermal exposure and spatial precision. In addition, scalable fabrication of complex multi-material systems remains technically demanding, frequently resulting in variability in mechanical and biological performance. Future research should emphasize closer integration of materials science with cell and developmental biology, advancing deployable, in situ, and injectable 4D systems to enable personalized, controllable, and dynamically adaptive therapies for bone and cartilage repair.

### 3.2. Muscle Regeneration: Structural Alignment and Dynamic Functional Adaptation

Skeletal and cardiac muscle are highly dynamic functional tissues that not only provide mechanical force generation but also rely on coordinated electrical signaling, vascular integration, and anisotropic structural organization for proper function. Following injury, effective muscle regeneration therefore requires multidimensional reconstruction, encompassing active contraction, electrical conductivity, and aligned fiber architecture [96]. Conventional 3D-printed homogeneous scaffolds often fail to replicate the highly ordered alignment and anisotropic mechanical behavior of native muscle fibers, limiting their ability to support functional recovery [97]. From a bio-inspired application perspective, 4D printing addresses these limitations by enabling constructs that combine controlled structural organization with adaptive, stimulus-responsive functionality, thereby overcoming the biomechanical constraints of static fabrication strategies.

#### 3.2.1. Macro- and Micro-Structural Alignment of Muscle Fibers

**Figure 2:**
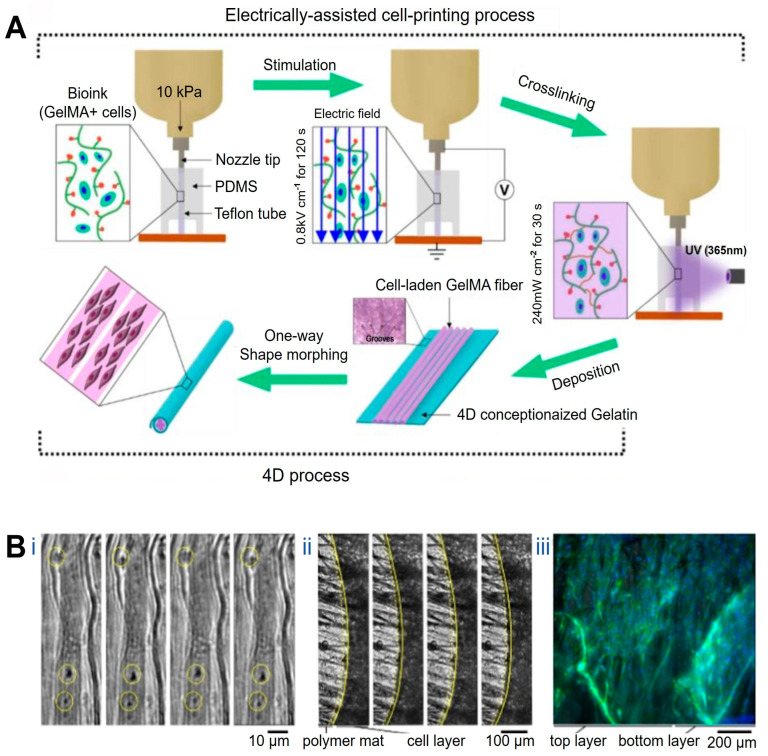
Applications of 4D printing in muscle regeneration. (**A**) Schematic describing electrically assisted cell-printing/crosslinking process and one-way shape morphing (4D) process for the fabrication of muscle fiber-like structures. Adapted from [99], under a CC-BY 4.0 licence. (**B**) Contractility of the muscle fibers layer under electrical stimulation (4−5 V, frequency: 1 Hz, duration: 1 ms): (**Bi**) functional contracting myotubes that are observed by cyclical displacement of features inside yellow circles, (**Bii**) contracting cell monolayer, solid and yellow dashed lines show edge of contracted and relaxed myotubes layer, respectively, (**Biii**) Time between images is 1 s; 3D projection of myoblast muscle cells on self-folded bilayer. Actin filament and nuclei staining using DAPI (blue) and Phalloidin (green) to evaluate the cell alignment on bilayer mats. Adapted from [100], under a CC-BY 4.0 licence.

At the structural level, 4D printing has been employed to recreate the hierarchical alignment characteristic of native muscle tissue. Constructs with precisely oriented microarchitectures have been fabricated using microgroove arrays of tunable widths in combination with near-infrared (NIR)-responsive materials such as shape-memory polymers and graphene, yielding myocardial models with highly ordered microstructures and adjustable curvature [98]. Upon NIR irradiation, these constructs undergo planar-to-curved transformation, reproducing aspects of myocardial geometry while supporting aligned cardiomyocyte growth and functional maturation with favorable biocompatibility and mechanical properties. In a complementary approach, gelatin methacrylamide (GelMA) bioinks loaded with C2C12 myoblasts have been printed under electric-field assistance, enabling field-induced cellular alignment during deposition. Subsequent UV crosslinking stabilizes the aligned hydrogel fibers, which can be assembled into bundled architectures resembling native muscle fascia (Figure 2A) [99].

#### 3.2.2. Dynamic Functional Adaptation Under External Stimuli

Beyond static structural alignment, 4D printing enables the construction of muscle constructs capable of dynamic, stimulus-induced deformation that approximates aspects of muscle motion. Gradient shape-change structures generated by modulating printing path angle and layer height during fused deposition modeling exhibit thermally actuated curling and unfolding behaviors. When fabricated using shape-memory polymers, these constructs form ordered, muscle-like arrangements that dynamically resemble skeletal muscle movements under thermal stimulation. Although such deformation does not fully replicate active muscle contraction, it demonstrates the feasibility of reconstructing motion-related morphology while maintaining cell viability and muscle-specific marker expression, supporting their applicability in muscle tissue engineering [101]. Similarly, bilayer constructs composed of polycaprolactone (PCL) microfiber meshes and methacrylated sodium alginate undergo spontaneous curling in aqueous environments to form “rolled-up muscle bundle”-like structures. When seeded with C2C12 cells and subjected to low-frequency electrical stimulation (1 Hz), these systems exhibit active contraction behavior (Figure 2B), representing an important step toward recreating muscle-like dynamic actuation in 4D-printed platforms [100].

In muscle regeneration, the distinctive advantage of 4D printing lies in its ability to integrate structural alignment with adaptive functional modulation, enabling constructs to be dynamically trained, remodeled, and adjusted over time in a manner analogous to native muscle tissue. Current strategies mainly include self-curling or gradient-strain scaffolds that promote long-range fiber alignment through spontaneous morphological adaptation, as well as the incorporation of conductive or piezoelectric components to introduce electrically triggered rhythmic actuation and accelerate muscle fiber maturation. In addition, injectable magnetically responsive hydrogels have emerged as a minimally invasive approach, forming anisotropic fiber networks in vivo under magnetic fields and significantly improving alignment in large defect models while reducing secondary surgical trauma [102]. Despite these advances, most reported contraction-like behaviors remain limited to passive deformation or partial synchronization, falling short of physiological force output, frequency responsiveness, and fatigue resistance. Interactions with nerves and vasculature are also frequently underrepresented, and the in vivo application of external fields raises concerns regarding safety, dose control, and penetration depth. Nevertheless, continued progress in injectable magnetic systems, conductive printing networks, and intelligent rehabilitation technologies is expected to advance 4D printing toward clinical translation for large-volume muscle repair and personalized regenerative therapies.

### 3.3. Vascular Regeneration: Programmable Morphogenesis and Dynamic Lumen Formation

Blood vessels are not merely tubular conduits but hierarchically organized, composite tissues composed of endothelial, smooth muscle, and adventitial layers, each contributing distinct yet interdependent mechanical and biological functions [103]. In vivo, native vasculature is continuously subjected to cyclic stretching, twisting, and bending under pulsatile flow, requiring dynamic adaptability to maintain structural integrity and physiological function. Consequently, static scaffolds often fail to recapitulate these behaviors. Although conventional 3D printing has enabled the fabrication of diverse vascular constructs, reproducing the intrinsic dynamic architecture and adaptive functionality of native vessels remains challenging [104,105]. From a bio-inspired application perspective, 4D printing offers a paradigm shift in vascular regeneration by enabling programmable morphological transformation, dynamic lumen formation, and construction of adaptive vascular microenvironments.

#### 3.3.1. Dynamic Structural Adaptation in Vascular Morphogenesis

**Figure 3:**
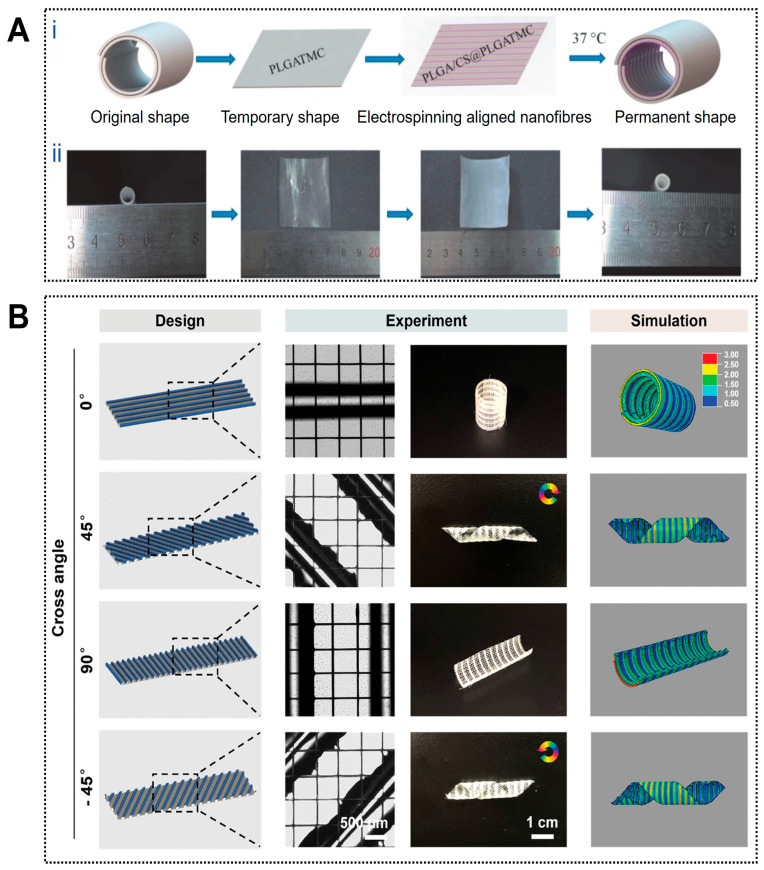
Applications of 4D printing in vascular regeneration. (A) Fabrication and programmed deformation of the PLGA/CS@PLGA-TMC scaffold. (**Ai**) Schematic illustration of the fabrication and the programmed deformation of PLGA/CS@PLGATMC scaffold and (**Aii**) corresponding photographs. Adapted with permission from Ref. [106]. Copyright 2020, Wiley−VCH. (**B**) The vein patterns of the paralleled hydrogel fibers directly impact the curling behavior of the Janus scaffolds upon dehydration. The hydrogel fibers in the active layer were oriented at an angle θ relative to the long axis of the structures. The initial pattern design (left column), experimental results (middle column), and the corresponding simulation (right column) of the Janus scaffolds with differently aligned hydrogel patterns. The rotation arrows indicate the direction of the helix tubular constructs and the color bar indicates the level of stress. Adapted with permission from Ref. [109]. Copyright 2024, Wiley−VCH.

4D printing enables controllable structural dynamics that closely approximate the deformation behavior of natural blood vessels. For example, a circumferentially aligned electrospun nanofiber membrane combined with an outer thermally responsive shape-memory polymer layer was designed to form a self-curling vascular construct capable of temperature-triggered transformation from a planar sheet into a cylindrical tube, thereby improving implantation operability (Figure 3A) [106]. Similarly, planar patterns of photo-crosslinked alginate dialdehyde/gelatin (Alg-dialdehyde/Gel) hydrogels were predesigned according to parameters such as tube diameter, folding angle, and layer thickness, enabling autonomous folding into predetermined tubular or T-shaped geometries upon hydration, with deformation kinetics precisely regulated by UV irradiation time [107]. Trujillo Miranda et al. further reported a bilayer electrospun system in which an outer thermoplastic fiber layer (PHB or PCL) provided mechanical support, while an inner hydrophilic HA-MA hydrogel layer enabled rapid swelling-driven deformation. By modulating fiber orientation, programmable crimping behaviors ranging from transverse to longitudinal modes were achieved, yielding constructs with favorable mechanical stability and biological compatibility [108].

#### 3.3.2. Microstructural Regulation for Cell Response

4D printing facilitates the generation of biomimetic microstructures that enhance endothelial adhesion, alignment, and tissue regeneration. Photosensitive hydrogels have been used to fabricate planar microarchitectures with spatially varied cross-linking densities through patterned illumination. Upon hydration, differential swelling generated localized strain fields that drove self-folding into complex three-dimensional vascular-like geometries, within which encapsulated endothelial cells proliferated, maintained physiological morphology, and preserved functional characteristics [110]. In another example, circumferentially aligned PLGA/chitosan electrospun inner layers promoted smooth muscle cell adhesion and orientation, contributing to mechanical anisotropy when integrated with an outer shape-memory polymer layer and further supporting vascular-like functionality [106].

#### 3.3.3. Construction of Multi-Branched and Hierarchical Vascular Networks

Importantly, 4D printing also enables the fabrication of highly intricate, branched, and multiscale vascular architectures that exceed the geometric limitations of static printing. Janus-structured scaffolds composed of a passive polycaprolactone layer and an active methacrylated gelatin/alginate hydrogel layer were programmed to undergo multistep shape transformations, including primary two-dimensional-to-three-dimensional tubular formation and secondary adaptation to local intravascular geometries after implantation (Figure 3B) [109]. Such dynamically programmable scaffolds enable the construction of perfusable constructs with tailored curvature and branching complexity, highlighting the potential of 4D printing for engineering multiscale vascular networks.

Overall, the integration of 4D printing into vascular regeneration enables dynamic morphological behaviors—including autonomous folding, curling, and branching—that more closely approximate the hierarchical and multibranched architecture of native vasculature. These capabilities improve implantation precision, reduce surgical complexity, and provide promising strategies for personalized reconstruction of small-diameter and multi-branch vessels. However, most current studies remain confined to in vitro systems or early animal models, and several challenges continue to hinder clinical translation. Key issues include insufficient validation of long-term stability and functional durability, limited reproducibility across complex vascular geometries, and variability in the biocompatibility, degradation behavior, response kinetics, and deformation accuracy of stimulus-responsive materials. In addition, printing resolution and mechanical strength often remain inadequate for sustained exposure to high-flow hemodynamic conditions, while heterogeneous in vivo environments—such as temperature gradients, fluid composition, and mechanical stresses—further complicate stimulus controllability. Future research should prioritize the integration of intelligent design strategies with multifunctional materials, combining digital modeling, finite element–based predictive shaping, and multimaterial printing to enable a transition from structural imitation toward clinically viable functional vascular reconstruction.

### 3.4. Neural Regeneration: Dynamic Guidance and Electrophysiological Functional Reconstruction

Peripheral nerve injury is a common clinical condition, and its incidence continues to increase, leading to neuralgia, motor dysfunction, and sensory deficits that severely impair patients’ quality of life [111]. Artificial nerve grafting has emerged as an important alternative for peripheral nerve and spinal cord repair. With advances in additive manufacturing, 3D-printed neural conduits, chips, and patches have been extensively explored, offering new possibilities for nerve repair [112,113]. However, most conventional scaffolds remain geometrically static and poorly adaptable to the evolving injury microenvironment. From a bio-inspired application perspective, 4D-printed neural scaffolds introduce dynamic responsiveness and adaptive reconstruction, enabling spatially guided nerve regeneration while maintaining favorable biocompatibility.

#### 3.4.1. Adaptive Morphogenesis and Self-Enclosing Neural Conduits

**Figure 4:**
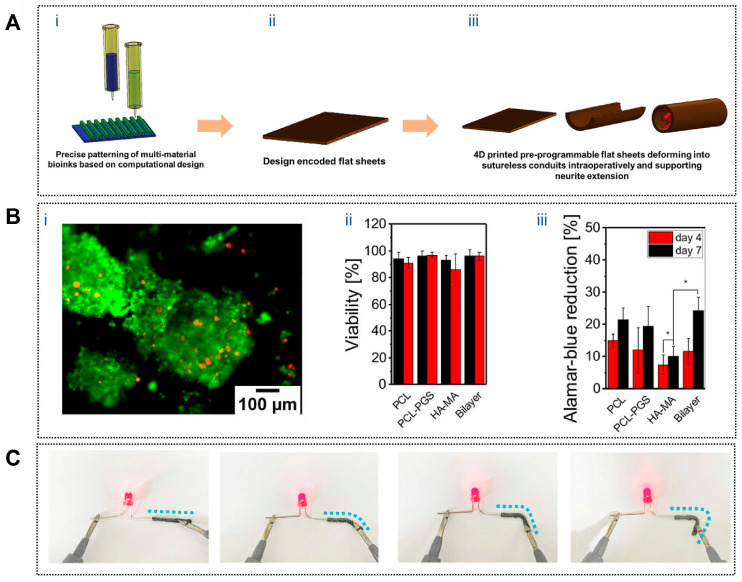
Applications of 4D printing in neural regeneration. (A) Process of fabrication of 4D printed nerve conduits with intraoperative shape deformation capability. (**Ai**) 3D printing of multi-material bioinks with precise patterning based on computational design. (**Aii**) 3D printed design encoded flat hydrogel sheets. (**Aiii**) Intraoperative deformation of the hydrogel sheet into nerve conduits, demonstrating the capability for neurite extension and nerve regeneration. Adapted with permission from Ref. [115]. Copyright 2023, Wiley-VCH. (B) PC-12 cell viability and proliferation on fibrous mats. (**Bi**) PC-12 neuron cells on PCL-PGS/HA-MA scaffold (Live-Dead assay that utilizes Calcein AM (green) (for live cells) and ethidium homodimer-1 (red) (for dead cells)); (**Bii**) quantification of the PC-12 neuron cell viability on fibrous scaffolds after 4 and 7 d of culture; (**Biii**) neuron cell proliferation on fibrous mats after 4 and 7 d of culture measured by Alamar Blue Assay (* *p* < 0.05). Adapted from [116], under a CC-BY 4.0 licence. (**C**) Photos of MXPLT lighting up an LED in straight and different bending states, indicating that it has good electrical conductivity. Adapted with permission from Ref. [117]. Copyright 2024, Wiley-VCH.

To emulate the enclosure provided by native nerve sheaths during repair, 4D-printed neural conduits have been designed to undergo autonomous shape transformation during or after implantation. A representative example involves a graphene nanosheet–reinforced composite that enables a planar scaffold to self-wrap into a tubular conduit upon exposure to water or heat, allowing adaptive encapsulation of the injured nerve without complex suturing procedures [114]. Similarly, a mixed alginate–methyl cellulose hydrogel sheet (Figure 4A) was engineered to self-roll in vivo, forming a seamless conduit that bridges transected sciatic nerve stumps. In a 45-day rat sciatic nerve defect model, this self-folding, sheath-like construct significantly enhanced nerve regeneration and achieved functional recovery comparable to or exceeding that of conventional sutured repair [115].

#### 3.4.2. Microstructural Guidance for Directed Axonal Growth

While adaptive enclosure establishes a permissive regenerative space, successful nerve repair further depends on the precise guidance of axonal extension along aligned pathways. This requirement has been addressed through dynamically formed anisotropic microarchitectures in 4D-printed scaffolds. Apsite et al. developed a bilayer electrospun conduit composed of an aligned polycaprolactone–polyglycerol succinate (PCL–PGS) fiber layer and a randomly oriented methacrylated hyaluronic acid (HA-MA) layer. Upon hydration, the bilayer spontaneously curled into a tubular structure with a tunable diameter (Figure 4B), while the aligned inner fibers provided effective contact guidance for PC12 cells, promoting oriented neurite outgrowth and directional nerve regeneration [116]. This dynamic formation of guidance channels closely reflects the structural cues present in native regenerating nerves.

#### 3.4.3. Bioelectrical Microenvironment Reconstruction for Functional Recovery

Beyond structural enclosure and directional guidance, restoration of the electrophysiological microenvironment is essential for functional neural regeneration. To address this requirement, shape-memory polymer conduits have been combined with conductive MXene nanosheets (Ti_3_C_2_T_x_) to construct systems capable of rapid self-rolling at physiological temperature (37 °C), enabling intraoperative self-wrapping (Figure 4C). Internal microchannels supported directional neural cell migration, while MXene-induced conductivity facilitated electrical signal transmission, partially reproducing the endogenous bioelectrical environment of peripheral nerves. In vivo studies demonstrated significantly improved muscle tension, motor performance, and nerve conduction velocity compared with nonconductive controls, with regenerative outcomes comparable to or exceeding those achieved with autologous nerve grafts [117].

Collectively, these bio-inspired 4D-printed neural scaffolds integrate adaptive enclosure, directional axonal guidance, and electrophysiological modulation to address multiple biological requirements of nerve regeneration simultaneously. Compared with fixed-form conduits, such systems reduce surgical invasiveness, dynamically conform to injury sites, and provide spatiotemporally coordinated cues that support axonal elongation and functional recovery. Nevertheless, challenges remain in ensuring predictable deformation under complex in vivo conditions and in evaluating the long-term biocompatibility, degradation behavior, and immunological safety of conductive nanomaterials. Moreover, most current evidence is derived from small-animal models, and translation to long-gap or large-diameter human nerves requires further validation. With the continued convergence of materials science, intelligent manufacturing, and neurobiology, bio-inspired 4D-printed neural scaffolds are expected to progress toward clinically viable solutions for precision, minimally invasive, and personalized nerve regeneration.

### 3.5. Wound Closure: Dynamic Contraction and Microenvironment-Responsive Regulation

Wound healing is a highly dynamic and staged biological process involving immediate wound contraction, inflammation control, angiogenesis, and tissue remodeling, all of which are tightly regulated by local microenvironmental cues and systemic conditions. Conventional wound dressings and static 3D-printed constructs primarily function as passive barriers and therefore struggle to coordinate with these evolving processes. In contrast, 4D-printed dressings are increasingly explored as bio-inspired wound management systems that actively participate in healing by mimicking natural wound contraction, responding to pathological microenvironmental signals, and providing real-time feedback on wound status. Through adaptive deformation, environment-triggered therapeutic delivery, and integrated sensing, 4D dressings enable a shift from passive coverage toward dynamic, process-oriented wound closure [118,119,120,121,122].

#### 3.5.1. Bio-Inspired Dynamic Contraction for Active Wound Closure

**Figure 5:**
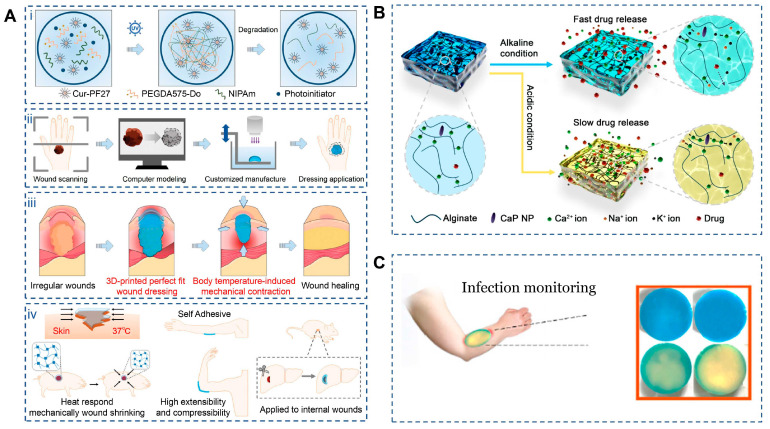
Applications of 4D printing in wound closure. (A) Schematic illustration of C-PNPD hydrogel wound dressing. (**Ai**) C-PNPD hydrogel is formed by mixing NIPAm, cur-PF127, PEGDA575-Do and Li-TMPP through photoinitiated free radical polymerization, and the formed network degrades in vivo as the wound healing process due to the cleavage of PEGDA575-Do crosslinker. (**Aii**) The conceptual design of the customized hydrogel patch for precise wound recognition and treatment, which consists of wound scanning, computer modeling, and customized manufacture (3D) printing. (**Aiii**) Programmable hydrogel dressing via wound recognition and 3D printing technology capable of adapting to the shape, size and depth of skin wounds or internal wounds. (**Aiv**) Scheme of body temperature-triggered wound shrinking by C-PNPD hydrogel wound dressing; the self-adhesive wound dressing can withstand high tensile and compressive forces, suitable for wound healing in joints; Application of hydrogel wound dressing for internal wounds. Adapted with permission from Ref. [123]. Copyright 2024, Wiley-VCH. (**B**) Schematic diagram of how CaP NPs can control the degradation of alginate dressings under different pH conditions. Adapted from [125], under a CC-BY 4.0 licence. (**C**) Smart multi-layer PVA foam/CMC mesh dressing with real-time infection monitoring. Adapted from [126], under a CC-BY 4.0 licence.

One of the most prominent bio-inspired features of 4D-printed dressings is their ability to reproduce wound contraction, a critical early event in physiological healing that reduces wound area and mechanical tension. Shape-memory and thermoresponsive materials allow dressings to autonomously conform to irregular wound geometries and actively contract under physiological conditions. Using digital light processing, Lu et al. fabricated composite dressings based on NIPAm/Cur-PF127/PEGDA575-Do that precisely matched wound topography (Figure 5A). The thermoresponsive N-isopropylacrylamide hydrogel underwent a phase transition at body temperature, inducing volumetric contraction that tightened the dressing around the wound surface, increased adhesion density, and reduced peripheral tension. Meanwhile, PEGDA575-Do provided strong tissue adhesion and controlled biodegradation, enabling stable fixation without traumatic removal and minimizing interference with healing progression [123]. Similarly, Sun et al. developed a biodegradable aliphatic polycarbonate elastomer (mPEG113-b-PMBCₙ) exhibiting rapid shape recovery at body temperature and intrinsic self-healing capability. Upon deployment, the material actively approximated wound edges within seconds and restored damaged regions over time, achieving superior closure performance compared with traditional sutures while maintaining favorable biocompatibility and degradability [124].

#### 3.5.2. Microenvironment-Responsive Regulation for Infection Control and Tissue Repair

In addition to mechanical contraction, effective wound healing relies on stage-dependent biochemical regulation, particularly in infected or chronic wounds. Bio-inspired 4D dressings address this requirement by sensing pathological microenvironmental changes—such as pH elevation or infection-associated thermal signals—and adjusting therapeutic release accordingly. pH-responsive alginate dressings fabricated via 3D printing demonstrate accelerated degradation and increased porosity under alkaline conditions typical of infected wounds, thereby enhancing the release of encapsulated antimicrobial agents while remaining relatively inert under physiological pH [127]. Shen et al. further designed a gelatin/oxidized dextran hydrogel incorporating copper peroxide (Figure 5B), which softened and partially swelled near body temperature to initiate release. Under near-infrared irradiation, photothermal conversion amplified copper ion and hydrogen peroxide generation, enhancing antibacterial efficacy. Importantly, infection-induced alkalinity accelerated network expansion and release, while normalization of pH during healing slowed delivery, reducing excessive tissue irritation. In parallel, copper ions promoted angiogenesis and tissue regeneration, collectively accelerating wound repair [125].

#### 3.5.3. Integrated Sensing and Real-Time Feedback During Wound Healing

Another hallmark of physiological wound healing is continuous monitoring of tissue status and timely adjustment of repair strategies. Mimicking this feedback mechanism, 4D-printed dressings have been developed with integrated sensing and visualization capabilities. Infection-associated biochemical changes—such as pH shifts, reactive oxygen species accumulation, and bacterial metabolites—can alter the optical properties of methylene blue, producing visible color changes that directly reflect wound condition. He et al. constructed a multilayer PVA/CMC dressing in which methylene blue was embedded in the PVA foam layer, which rapidly interacted with wound exudates (Figure 5C). The highly absorptive PVA facilitated fast signal response, while the CMC mesh ensured uniform exudate distribution. Upon photodynamic activation, methylene blue generates singlet oxygen, enhancing antibacterial activity and intensifying colorimetric contrast, enabling real-time, instrument-free monitoring suitable for both clinical and home-based wound management [126].

Taken together, 4D-printed dressings represent a bio-inspired evolution in wound closure strategies by actively emulating key healing behaviors rather than serving as passive coverings. Through programmable contraction, environment-adaptive therapeutic delivery, self-healing and degradable architectures, and real-time feedback, these systems reduce secondary interventions while improving healing efficiency. Nonetheless, challenges remain in optimizing response speed, maintaining long-term structural and functional stability, and achieving coordinated multi-stimulus responsiveness, particularly in large or complex wounds. Future efforts should focus on developing highly sensitive, programmable materials and exploring in situ or injectable 4D printing approaches to realize personalized, dynamic, and sustainable wound management, thereby accelerating clinical translation.

### 3.6. Other Biomedical Applications: Programmable Deployment and Physiological Adaptation

In addition to musculoskeletal, vascular, neural, and cutaneous tissues, 4D printing has been increasingly applied to a range of emerging biomedical scenarios in which anatomical complexity, physiological motion, and minimally invasive deployment impose stringent design requirements. Across these diverse applications, a common bio-inspired theme lies in enabling implants or constructs to reversibly transition between compact delivery states and functional configurations that conform to dynamic biological environments.

**Figure 6:**
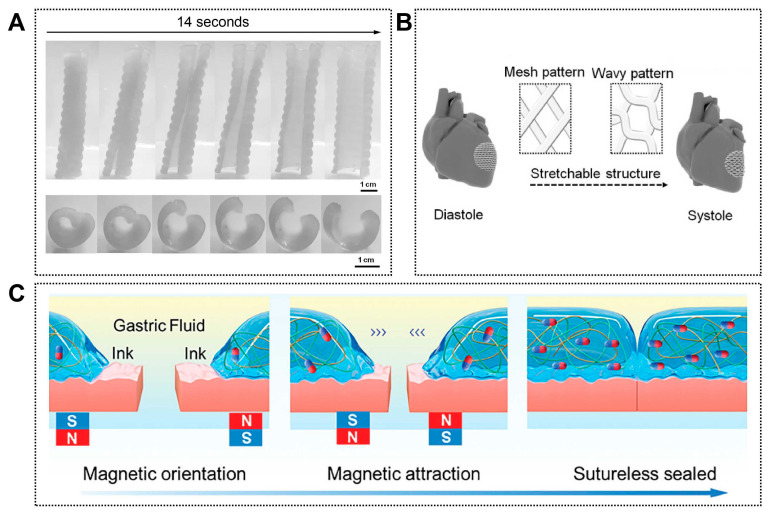
Other representative emerging applications of 4D printing. (**A**) Photoseries of macroscopic shape memory behavior of a personalized airway stent. Adapted with permission from Ref. [128]. Copyright 2017, Wiley-VCH. (**B**) CAD design of a 3D stretchable architecture of the heart. It provides dynamic stretchability without material deformation or failure when the heart repeatedly contracts and relaxes. Adapted from [130], under a CC-BY 4.0 licence. (**C**) Schematic illustration of stomach sutureless sealing controlled by an external magnetic field. Adapted with permission from Ref. [129]. Copyright 2024, Wiley-VCH.

In airway reconstruction, Zarek et al. employed ultraviolet-cured stereolithography to fabricate patient-specific tracheal constructs using shape-memory methacrylated polycaprolactone (PCL) prepolymers. After thermal programming, the devices could be compressed into planar or compact forms to facilitate minimally invasive insertion and subsequently recover their predefined three-dimensional geometry at body temperature, achieving close anatomical conformity within the tracheal lumen (Figure 6A). This approach effectively mimics the deployable yet resilient behavior required of endoluminal airway supports and demonstrates the integration of imaging-based customization, smart polymer design, and high-precision additive manufacturing [128].

A similar bio-inspired deployment strategy has been explored for gastrointestinal repair. Shi et al. developed magnetically responsive polyelectrolyte composite hydrogels that could be delivered endoscopically and remotely guided to defect sites using external magnetic fields, enabling suture-free closure of gastric perforations (Figure 6C). By emulating targeted navigation and in situ adaptation within confined luminal spaces, this strategy highlights how 4D-printed systems can interface with clinical guidance technologies to achieve precise and atraumatic tissue repair [129].

Dynamic mechanical responsiveness has also been exploited in cardiac regeneration, where continuous cyclic loading plays a critical role in tissue maturation. Cui et al. designed a physiologically adaptive 4D cardiac patch capable of providing mechanically active cues that approximate native myocardial motion (Figure 6B). Under physiological stimulation, the patch promoted vascularization and cardiomyocyte maturation, illustrating a bio-inspired shift from static cardiac supports toward mechanically intelligent implants for myocardial repair [130].

In orthodontics, the oral environment presents a distinct yet equally demanding setting characterized by confined space, continuous mechanical loading, and thermal fluctuations. Atta et al. introduced photocurable shape-memory polymers to fabricate temperature-responsive orthodontic aligners capable of controlled shape recovery under intraoral conditions or brief external heating. These aligners delivered stable and programmable orthodontic forces while reducing the frequency of appliance replacement, reflecting a bio-inspired strategy that aligns therapeutic force delivery with physiological conditions [131].

Together, these examples demonstrate that the value of 4D printing in emerging biomedical applications lies not in any single tissue type, but in its ability to emulate key physiological behaviors—such as deployability, conformability, mechanical adaptability, and environment-driven functionality—across diverse organ systems. By transcending the limitations of static constructs, 4D-printed systems offer new opportunities for personalized, minimally invasive, and functionally adaptive therapies. As advances in smart material chemistry, printing precision, and image-guided or endoscopic delivery continue to converge, 4D printing is expected to play an increasingly important role in the development of next-generation bio-inspired medical devices and regenerative solutions.

## 4. Challenges and Future Perspectives

The defining feature of 4D printing lies in the incorporation of time-dependent adaptability into additively manufactured constructs, enabling programmed structural or functional evolution under external stimuli. While this capability has opened new avenues for engineering biomimetic tissues with enhanced dynamic relevance, current progress remains largely confined to proof-of-concept studies and small-animal models. This disparity highlights a fundamental gap between engineered responsiveness and the multifactorial, spatiotemporally regulated nature of native biological systems. To facilitate meaningful clinical translation, it is therefore essential to distinguish the challenges that require immediate resolution from those representing near-term research priorities, longer-term technological development, and eventual translational implementation.

### 4.1. Immediate Challenges: Material Performance and Biological Compatibility

At present, the most critical bottleneck in bio-inspired 4D printing arises from the limited ability of available smart materials to simultaneously satisfy mechanical integrity, biological compatibility, and adaptive responsiveness. Although shape-memory polymers, responsive hydrogels, and temperature-, light-, or magnetic-sensitive systems have substantially expanded the design toolbox, their performance often falls short when applied to complex biological contexts. This limitation becomes particularly apparent in multicellular co-culture systems, hierarchically organized tissues, and constructs exposed to sustained mechanical loading, where compromises between print fidelity, responsiveness, and long-term bioactivity are frequently unavoidable [33,132]. In contrast to native tissues—where mechanical stability, biochemical signaling, and adaptive remodeling are tightly coordinated—most existing 4D-printed constructs rely on single-stimulus activation, offering limited capacity to emulate the spatially heterogeneous and dynamically regulated physiological microenvironment [102]. Addressing this mismatch represents an immediate priority, necessitating the development of biodegradable, multi-responsive material systems capable of adapting to tissue-specific cues through coordinated structural and biochemical regulation.

### 4.2. Near-Term Priorities: Coupling Deformation with Biological Regulation

Beyond material performance, a major near-term challenge lies in clarifying how programmed deformation influences cellular behavior and tissue development. Current studies predominantly focus on macroscopic shape transformations, such as bending or unfolding, as primary indicators of 4D functionality. However, the downstream biological consequences of these dynamic changes—particularly their effects on cell migration, alignment, differentiation, and mechanotransduction—remain insufficiently characterized [28,133,134]. In living tissues, mechanical signals are inseparably linked to biochemical pathways that guide morphogenesis and functional maturation. By comparison, deformation in many 4D-printed constructs is treated as a geometric endpoint rather than as an instructive biological signal. Bridging this gap will require systematic, multiscale investigations into cell–matrix interactions and force-mediated signaling during dynamic transformation, supported by integrated multi-physics and multi-scale modeling approaches to enable predictive and biologically informed design.

### 4.3. Mid- to Long-Term Development: Intelligent and Spatiotemporally Controlled Fabrication

As research progresses toward greater biological complexity, limitations in fabrication technologies increasingly constrain achievable construct fidelity and functionality. The generation of branched vascular networks, neural architectures, or spatially heterogeneous composite tissues demands printing platforms capable of microscale resolution, precise multi-material coordination, and temporally programmable control [135]. However, commonly employed techniques—including extrusion-based, inkjet, and photopolymerization printing—each exhibit inherent trade-offs when applied to dynamically transformable systems. Over the mid to long term, advances are expected to arise from the integration of intelligent manufacturing strategies, such as synchronized multi-channel printing, real-time optical or magnetic feedback, and AI-assisted adaptive path planning. Together, these approaches may enable the fabrication of personalized constructs with tightly regulated spatiotemporal behavior and higher-order structural complexity.

### 4.4. Translational Outlook: Evaluation Frameworks and Clinical Readiness

Despite substantial technological progress, translational barriers remain a decisive factor limiting clinical adoption. Most in vivo evaluations of 4D-printed constructs rely on small-animal models, offering limited insight into long-term biocompatibility, degradation dynamics, and functional stability under clinically relevant conditions [136,137,138]. Moreover, the dynamic and evolving nature of 4D-printed systems challenges conventional assessment paradigms established for static implants. From a regulatory standpoint, such constructs occupy an ambiguous position at the intersection of medical devices, tissue-engineered products, and intelligent systems, complicating standardization and approval. The establishment of dedicated evaluation frameworks addressing responsiveness accuracy, long-term safety, and functional evolution will therefore be essential, requiring coordinated efforts among researchers, industry stakeholders, and regulatory bodies.

Overall, the adaptive and programmable nature of 4D printing aligns closely with the goals of personalized and precision regenerative medicine. The convergence of image-based modeling, AI-assisted design, and digitally guided fabrication offers a conceptual pathway toward patient-specific constructs capable of dynamic biological integration. Ultimately, progress in this field will depend not only on advances in individual technologies but also on interdisciplinary collaboration across materials science, engineering, biomedicine, and clinical research. Such coordinated efforts will be crucial for transforming bio-inspired 4D printing from an experimental platform into a clinically viable regenerative strategy.

## 5. Conclusions

As an emerging interdisciplinary field integrating intelligent materials science with precision manufacturing, 4D printing is progressively driving a paradigm shift in regenerative medicine and tissue engineering from static replacement toward dynamic regeneration. Its broad applicability across bone, vascular, muscle, and neural tissues highlights its unique capacity to support personalized, functional, and intelligent repair strategies. By enabling programmable adaptation in response to biological cues, 4D printing offers an unprecedented opportunity to better align engineered constructs with the dynamic nature of living tissues.

Nevertheless, the translation of 4D printing into clinical practice remains constrained by challenges related to material performance, manufacturing accuracy, biological response mechanisms, safety evaluation, and translational pathways. Addressing these issues will require sustained interdisciplinary collaboration, advances in high-performance smart materials, refinement of intelligent manufacturing systems, and the establishment of robust evaluation frameworks. Ultimately, the regenerative paradigm driven by 4D printing reflects a deeper attempt to recapitulate the dynamic essence of life itself. With the continued convergence of materials science, artificial intelligence, and medicine, 4D printing has the potential to redefine the boundaries of regenerative therapy, advancing from damage repair toward functional tissue reconstruction.

## Figures and Tables

**Table 1:** Overview of representative 4D-printing materials and their key characteristics.

Material Type	Representative Materials	Mechanistic Properties	Primary Stimuli-Responsive Mechanisms	Key Features	Typical Applications	Refs.
**Shape Memory Polymers (SMPs)**	Polyurethane (PU), Poly(ε-caprolactone) (PCL), Poly(butylene terephthalate) (PBT), Epoxy resins	Based on the cooperative action between rigid (fixed) and soft (reversible) segments, enabling chain rearrangement and reversible phase transition upon external stimuli.	Temperature (primary), light, electric field, pH (material- and composite-dependent)	Flexible processing, lightweight and ductile; tunable transition temperature and mechanical performance; multi-stimuli responsiveness; can be functionalized via nanofiller incorporation.	Self-expanding scaffolds, minimally invasive implants (stents, valves), temperature/pH-triggered drug delivery systems, and wearable medical devices.	[18,19,20,21,22,23,24,25,26,27,28,29,30]
**Hydrogels**	Gelatin methacrylate (GelMA), Poly(N-isopropylacrylamide) (PNIPAm), Alginate, Hyaluronic acid	Three-dimensional hydrophilic polymer networks that swell and form hydrated structures upon water absorption; undergo reversible shrinkage or swelling under stimuli.	Temperature, pH, light	Excellent biocompatibility and degradability; strong multi-stimuli responsiveness; suitable for co-loading cells/drugs; high printing resolution for complex geometries.	Smart wound dressings (pH-responsive drug release), bone/cartilage/vascular scaffolds, thermo-/photo-responsive drug delivery systems, soft robotics, and cell culture matrices.	[31,32,33,34,35,36,37,38]
**Liquid Crystal Materials (LCPs/LCEs)**	PMMA-based liquid crystalline elastomers, acrylate-siloxane copolymers, aromatic polyester LCPs	Macroscopic behavior governed by molecular alignment; exhibits large and reversible deformations under stimuli.	Temperature, light, electric field	LCPs: high strength, chemical resistance; LCEs: fast and reversible actuation; programmable molecular orientation; multi-stimulus response.	Artificial muscles, soft robotics, microscale actuators, flexible and wearable electronics.	[39,40]
**Shape Memory Alloys (SMAs)**	NiTi alloy, Cu-Al-Ni alloy, Fe-Mn-Si alloy	Reversible martensitic ↔ austenitic phase transformation; deformable and recoverable under stimuli, showing shape memory and superelasticity.	Temperature, mechanical stress	High strength and toughness; remarkable shape recovery; superior elasticity compared with polymers/hydrogels; capable of complex geometries.	Self-expanding vascular stents, orthopedic implants, surgical instruments, adaptive bone scaffolds.	[41,42,43,44,45,46]
**Shape Memory Ceramics (SMCs)**	Zirconia (ZrO_2_), Barium titanate (BaTiO_3_), Alumina-based composites	Based on reversible crystalline phase transitions under stimuli.	Temperature, mechanical stress (limited and system-dependent)	Excellent thermal stability, hardness, and wear resistance; maintains performance under extreme environments; can be composited for multi-stimulus response.	High-temperature smart devices, adaptive insulation/thermal barriers, micro-actuators and sensors; biomedical applications under early exploration.	[47,48]

**Table 2:** Overview of representative 4D-printing techniques and their key characteristics.

	Extrusion-Based		Spraying	Photopolymerization		Powder Bed Fusion	
	DIW	FDM	Inkjet	SLA	DLP	SLS	SLM
**Core Processing Principle**	Continuous extrusion of viscoelastic inks through a nozzle	Thermal melting and extrusion of thermoplastic filaments	On-demand ejection of discrete droplets via thermal or piezoelectric actuation	Point-wise photopolymerization using a focused light source	Layer-wise photopolymerization using projected light patterns	Laser-induced sintering of polymer powder beds	Full melting of metal powders using a high-energy laser
**Typical Materials**	SMPs, stimuli-responsive hydrogels, polymer–ceramic composites, bioinks	Thermoplastic SMPs (PLA, PCL, TPU), polymer composites	Low-viscosity polymers, functional inks, bioactive solutions	Photo-crosslinkable SMPs, hydrogels, LCE precursors	Photocurable polymers, hydrogels, shape-memory networks	Shape-memory polymers, medical-grade polymers	Shape-memory alloys (e.g., NiTi), biocompatible metals
**Key Advantages**	Broad material tolerance; easy introduction of anisotropy, gradients, and pre-strain	Simple setup; good mechanical strength; suitable for thermo-responsive SMP structures	High spatial resolution; precise local material placement	High resolution and surface quality; programmable crosslink density	Fast printing speed; uniform curing; effective for self-morphing structures	No support structures required; good mechanical integrity	Enables stress-/temperature-responsive metal 4D constructs; high strength
**Main Limitations**	Moderate resolution; shape fidelity depends on ink rheology	Limited to melt-processable materials; high temperature excludes cells	Strict viscosity and surface tension requirements	Limited material diversity; photoinitiator-related cytotoxicity concerns	Restricted to light-sensitive systems; limited multi-material capability	High processing temperature; limited biological integration	Extreme temperatures; post-processing required; unsuitable for soft tissues
**Typical Applications**	Shape-morphing scaffolds, self-folding hydrogels	Thermally activated shape-memory implants	Micro-patterned responsive structures	Self-folding constructs, programmable microstructures	Rapid fabrication of self-morphing architectures	Load-bearing polymeric 4D components	Self-expanding stents, adaptive orthopedic implants
**Refs**	[70,71,72]		[73,74]	[75,76]		[77,78]	

## Data Availability

No new data were created or analyzed in this study. Data sharing is not applicable to this article.
